# What Are We Measuring When We Evaluate Digital Interventions for Improving Lifestyle? A Scoping Meta-Review

**DOI:** 10.3389/fpubh.2021.735624

**Published:** 2022-01-03

**Authors:** Rodolfo Castro, Marcelo Ribeiro-Alves, Cátia Oliveira, Carmen Phang Romero, Hugo Perazzo, Mario Simjanoski, Flavio Kapciznki, Vicent Balanzá-Martínez, Raquel B. De Boni

**Affiliations:** ^1^Escola Nacional de Saúde Pública Sergio Arouca, Oswaldo Cruz Foundation (Fiocruz), Rio de Janeiro, Brazil; ^2^Instituto de Saúde Coletiva, Universidade Federal do Estado do Rio de Janeiro, Rio de Janeiro, Brazil; ^3^Instituto Nacional de Infectologia Evandro Chagas, Oswaldo Cruz Foundation (Fiocruz), Rio de Janeiro, Brazil; ^4^Centro de Desenvolvimento Tecnológico em Saúde, Oswaldo Cruz Foundation (Fiocruz), Rio de Janeiro, Brazil; ^5^Department of Psychiatry and Behavioural Neurosciences, McMaster University, Hamilton, ON, Canada; ^6^Bipolar Disorder Program, Laboratory of Molecular Psychiatry, Instituto Nacional de Ciência e Tecnologia Translacional em Medicina, Hospital de Clínicas de Porto Alegre, Porto Alegre, Brazil; ^7^Department of Psychiatry, Universidade Federal do Rio Grande do Sul, Porto Alegre, Brazil; ^8^Teaching Unit of Psychiatry and Psychological Medicine, Department of Medicine, University of Valencia, CIBERSAM, Valencia, Spain; ^9^Institute of Scientific and Technological Communication and Information in Health, Oswaldo Cruz Foundation (Fiocruz), Rio de Janeiro, Brazil

**Keywords:** digital health interventions, lifestyle, diet, physical activity, substance use, stress management, social relationship, sleep

## Abstract

**Background:** Lifestyle Medicine (LM) aims to address six main behavioral domains: diet/nutrition, substance use (SU), physical activity (PA), social relationships, stress management, and sleep. Digital Health Interventions (DHIs) have been used to improve these domains. However, there is no consensus on how to measure lifestyle and its intermediate outcomes aside from measuring each behavior separately. We aimed to describe (1) the most frequent lifestyle domains addressed by DHIs, (2) the most frequent outcomes used to measure lifestyle changes, and (3) the most frequent DHI delivery methods.

**Methods:** We followed the Preferred Reporting Items for Systematic Reviews and Meta-Analyses (PRISMA-ScR) Extension for Scoping Reviews. A literature search was conducted using MEDLINE, Cochrane Library, EMBASE, and Web of Science for publications since 2010. We included systematic reviews and meta-analyses of clinical trials using DHI to promote health, behavioral, or lifestyle change.

**Results:** Overall, 954 records were identified, and 72 systematic reviews were included. Of those, 35 conducted meta-analyses, 58 addressed diet/nutrition, and 60 focused on PA. Only one systematic review evaluated all six lifestyle domains simultaneously; 1 systematic review evaluated five lifestyle domains; 5 systematic reviews evaluated 4 lifestyle domains; 14 systematic reviews evaluated 3 lifestyle domains; and the remaining 52 systematic reviews evaluated only one or two domains. The most frequently evaluated domains were diet/nutrition and PA. The most frequent DHI delivery methods were smartphone apps and websites.

**Discussion:** The concept of lifestyle is still unclear and fragmented, making it hard to evaluate the complex interconnections of unhealthy behaviors, and their impact on health. Clarifying this concept, refining its operationalization, and defining the reporting guidelines should be considered as the current research priorities. DHIs have the potential to improve lifestyle at primary, secondary, and tertiary levels of prevention—but most of them are targeting clinical populations. Although important advances have been made to evaluate DHIs, some of their characteristics, such as the rate at which they become obsolete, will require innovative research designs to evaluate long-term outcomes in health.

## Introduction

According to the latest Global Burden of Disease Study, the drivers of increased Disability-adjusted life years (DALYs) from 1990 to 2019 include six health problems that mostly affect adults older than 50 years (such as ischemic heart disease and diabetes) and four that mostly affect individuals from 10 to 49 years (such as depressive disorders) (1). In 2019, eight of the top 10 risk factors for death and disability were behavioral or metabolic problems [such as high systolic blood pressure, smoking, high fasting plasma glucose, high body-mass index, high low-density lipoprotein (LDL) cholesterol, and alcohol use] (2). Such figures, in addition to the extensive literature on the matter (3–10), emphasize the importance of addressing unhealthy behaviors to prevent morbidity and mortality.

Lifestyle Medicine (LM) profits from evidence-based strategies to prevent and treat the progression of chronic diseases and improve quality of life (11). As per the American College of Lifestyle Medicine (12), the LM foundation is established over six main domains: diet, physical activity (PA), avoiding substance use (SU), sleep, social relationships, and stress management. Thus, LM aims toward a comprehensive change in unhealthy behaviors. Unhealthy behaviors tend to cluster and present an additive effect on decreased survival time without disability and earlier mortality (5, 8, 10, 13). However, most epidemiological studies do not include all the lifestyle domains considered in LM when evaluating health outcomes. Instead, unhealthy behaviors have been considered as independent risk factors. The multidimensional evaluation of lifestyle is becoming more frequent, either by using lifestyle indexes (5, 10) or including multiple lifestyle domains and testing their interaction effects (9). In this sense, our research group recently developed and validated a scale for the multidimensional evaluation of lifestyle (14–17).

Digital health interventions (DHIs) may be understood as interventions delivered *via* digital technologies (such as apps, digital platforms, and wearables) to improve the health of individuals (18–21). DHIs have increasingly been used to promote behavior change and a healthier lifestyle. For instance, a study conducted in Australia (June 2018–July 2018) searched for digital apps, using the words “health” and “well-being” (in the Australian iTunes and Google Play), and retrieved 2,12,352 related apps (22). In addition, DHIs have been advocated as a way to increase access to health, such as mental health (23), due to their relatively low cost and ease of scalability. However, the debate on how DHIs must be evaluated to ensure their quality and efficacy is far from over (24, 25). Due to the velocity and dynamic changes of DHIs, some argue that the gold standard of medical evaluation—randomized clinical trials (RCT)—would not be appropriate to DHIs assessment. Such velocity may also jeopardize the broader evaluation of effectiveness—which is usually made through systematic reviews and meta-analyses. Whenever there are multiple systematic reviews on the same topic it may be necessary to conduct a meta-review (i.e., the systematic review of systematic reviews) (26). Moreover, if the field is highly heterogeneous and/or wide, scoping meta-reviews may be necessary to summarize concepts, theories, sources, and knowledge gaps.

An important step in the advancement of DHI evaluation was the publication of the Evidence Standards Framework for Digital Health Technologies (27), in the United Kingdom (UK). Although developed to “demonstrate (DHIs) value in the UK health and social care system,” it may be useful for guiding other countries, especially those presenting universal healthcare systems. Regarding digital interventions for preventing unhealthy lifestyles, the guideline recommends a minimum of high-quality observational or quasi-experimental studies demonstrating relevant outcomes. The determination of relevant outcomes in LM, however, is not simple. In 2017, a meta-review on DHIs for cardiometabolic events pointed to the heterogeneity in study measures, such as DHI modalities, populations, and outcomes. Such heterogeneity precluded the formation of strong conclusions regarding the effectiveness of the evaluated interventions (28). In a meta-review evaluating the effect of DHIs on mental health, studies focusing on symptoms of anxiety or depression presented positive small/medium effect sizes (29). However, mental health symptoms are also heterogeneous and may not always represent a clinical psychiatric diagnosis, which could jeopardize future attempts to reproduce the findings.

Besides the outcomes, control groups also may represent a challenge. For instance, a meta-review evaluating DHIs for weight loss concluded that the interventions were more effective than minimal treatment but less effective than face-to-face interventions (30). Also, it is possible that some lifestyle domains, such as PA and diet, are of greater interest for addressing and evaluating DHIs due to the easily measurable outcomes associated with these domains. This hypothesis may make it even harder to evaluate lifestyle as a multidimensional construct, since easily measurable outcomes may lead to an unbalance in the field, with higher representation of lifestyle domains from which there is more available evidence.

Given the huge heterogeneity of DHIs and the specific challenges related to lifestyle research, in this article, we aimed to (1) identify the most frequent lifestyle domains evaluated (diet, PA, SU, sleep, social relationships, and stress management—as proposed by the American College of Lifestyle Medicine (12), and most common combinations of them); (2) describe the most frequent outcomes used to measure lifestyle changes in each domain or in multidimensional evaluations; and (3) to identify the most frequent delivery methods. Answering these questions and revealing research gaps is a crucial step for designing, implementing, and evaluating DHI in order for these interventions to have clinical and public health relevance.

## Methods

The methodology followed Arksey and O'Malley's framework for scoping reviews (31) and the Preferred Reporting Items for Systematic Reviews and Meta-Analyses Extension for Scoping Reviews (PRISMA-ScR) (32). A scoping review is defined as a type of research synthesis that aims to map the literature on a particular topic or research area to identify key concepts, research gaps, and sources of evidence to inform practice, policymaking, and research.

The review included the following key phases: (a) formulating a research question, (b) Population, Intervention, Comparator, Outcome, Study Design (PICOS) acronym and eligibility criteria definition (c) identifying relevant studies, (d) study selection, (e) data extraction, and (f) collating, summarizing, and reporting the results.

### Research Question

The main research question was, “What are we measuring when we evaluate digital interventions for improving lifestyle?” Specifically, we aimed to answer the following questions: “What are the most frequent lifestyle domains addressed by DHI?,” “What are the most frequent outcomes used to measure lifestyle changes?,” and “What are the most frequent DHI delivery methods?”

### Inclusion and Exclusion Criteria

We included systematic reviews or meta-analyses of RCTs that assessed the effectiveness of DHI focusing on any of the lifestyle domains proposed by the American College of Lifestyle Medicine (i.e., diet, SU, PA, social relationships, sleep, and stress management). Those reviews had to include adults (equal or above 18 years) either from the general population, patients, or at-risk population. DHIs could be delivered by smartphone apps, computer or tablet, digital games, digital platforms, monitoring devices, social media, websites, SMS, and/or e-mail. There were no restrictions regarding the control group. Reviews were included if they were published after 2010 (following the widespread use of smartphones) and if published in English, Portuguese, Spanish, or French.

Exclusion criteria were systematic reviews of observational or qualitative studies, study protocols, types of publications different from full articles, such as congress abstract, letters, or comments to the editor. Studies that include only children, adolescents, or pregnant women were excluded because outcomes evaluating the effectiveness of DHI may not be applied to adults (such as the growth curve or early childbirth). Studies including only interventions delivered by SMS were also excluded.

### Information Sources and Search Strategies

The search was carried out in September 2020 and updated in October 2021 across four electronic databases: MEDLINE through PubMed, Cochrane Library, EMBASE, and Web of Science. Construction of the search strategy was written using controlled vocabulary terms, specific to the databases; main descriptors were: “lifestyle,” “e-health,” “m-health,” and other similar terms, such as “systematic review” and “clinical trials.” Supplementary Material 1 presents the strategies for each bibliographic database. Additionally, the reference lists of the selected articles were manually scrutinized for other studies that could have been lost in the electronic search.

### Study Selection

After the removal of duplicated references, titles and abstracts were screened according to inclusion criteria. Full texts of articles meeting the inclusion criteria were retrieved and checked for their eligibility through complete reading. The process of screening citations and selecting articles was carried out independently by two reviewers, and the discrepancies were resolved by consensus or decided by a third researcher. A table containing the list of excluded studies along with the reasons for their exclusion was prepared (Supplementary Material 1). The Rayyan QCRI web application (https://rayyan.qcri.org/) (33) and Mendeley^®^ were used to screen and manage the references, respectively; both are open-access.

### Data Extraction

Data extraction was conducted independently by two reviewers using a pre-pilot spreadsheet in the Excel program. The developed spreadsheet was tested by the study team in a small sample of papers (*n* = 2) and calibrated before use. Discordances in the extracted data were resolved by consensus. Data extracted from each study are presented in Table 1.

**Table 1:** Data extracted from the studies.

**Item**	**Description/classification**
Author(s) and year of publication	Description of the authors and year of publication of the study
Lifestyle domains	• Diet • Substance use • Physical activity • Social relationships • Sleep • Stress management
Is there a meta-analysis	Yes/No
Population	• General population • Patients • At-risk population • All of the above
Number of RCT studies	Number of RCT studies included for analysis
Sample size	Range of participants; lowest and highest sample size
Delivery method of e-health intervention	• App/smartphone • Computer/tablet • Digital games • Digital platform • E-mail • Monitoring devices • Social media • Website
Comparison groups	• Control groups/usual care • Different modes of delivery • Other e-health intervention • All of the above
Outcome	• Objective behavior change • Scales and self-reported measures • Biochemical measures • Clinical conditions
Outcome description	Possible measures under each Outcome
Lifestyle scales used	Lifestyle scales used to answer the research question

### Data Analysis

The data were compiled into a single Excel 2010 spreadsheet for validation and coding. To avoid frequent issues related to papers using different terminology, before the analysis, all string data were reviewed. Synonyms were merged in unique terms decided by consensus with the reviewers. Descriptive statistics were performed analyzing the frequency of each lifestyle domain, the outcomes, and the delivery methods. In constructing the graphs, we used the software R v.4.0.5, the library Rgraphviz and its dependencies (34).

## Results

**Figure 1:**
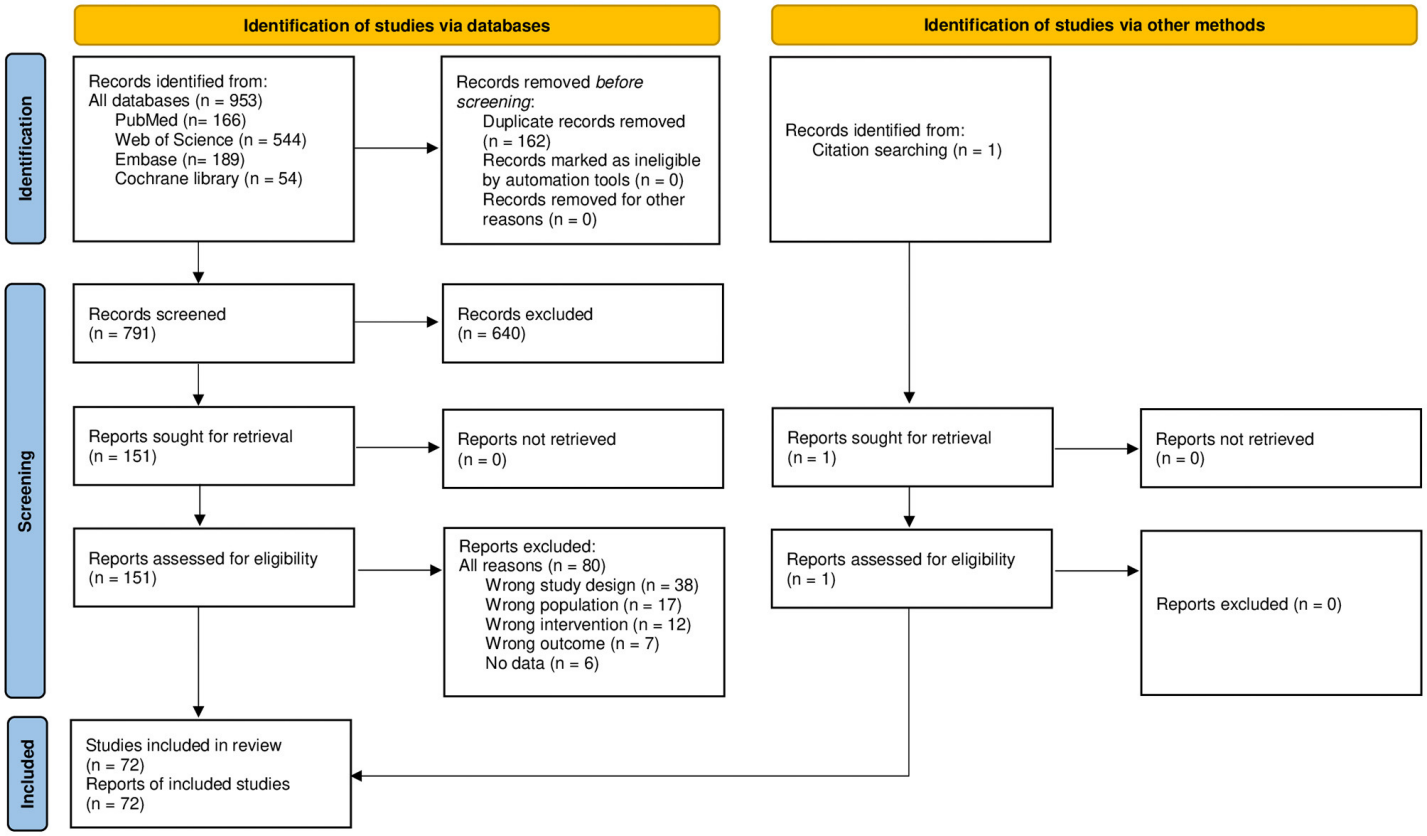
PRISMA 2020 flow diagram. PRISMA, the Preferred Reporting Items for Systematic Reviews and Meta-Analyses.

Overall, 953 records were identified by searching the databases, and 1 record was additionally found through the manual search. Title and abstract screenings resulted in 152 included systematic reviews or meta-analyses reporting on the effectiveness of DHIs focusing on one or more of the lifestyle domains evaluated (i.e., diet, SU, PA, social relationships, sleep, and stress management) among adults. A total of 72 studies were included after full-text assessment, of which 35 conducted meta-analyses (Figure 1). All of them were published in English.

The systematic reviews included data ranging from a total of 2 (35) to 117 (36) RCTs. Overall, the sample size of the included RCTs ranged between 6 (37) and 69,219 (38) participants. Unfortunately, some systematic reviews did not report the size of the included RCTs. Most DHIs were compared to more than one alternative strategies. Table 2 shows the summary of systematic reviews included.

**Table 2:** Summary of included systematic reviews, 2010–2021.

**References**	**Population**	**# RCT**	**Size range**	**Delivery method**	**Comparison group**	**Meta-analysis**	**Domain**
Akinosun et al. (39)	All	25	44–710	app, PC, mon dev	SoC	Yes	Diet, SU, PA
Allen et al. (40)	Patients -overweight/obese	39	51–2,862	dig plat, website	>1	No	Diet
Beleigoli et al. (41)	At-risk -overweight/obese	11	35–440	app, dig plat, email, website	>1	Yes	Diet, PA
Beratarrechea et al. (42)	Patients (CD)	9	16–225	app, email	>1	No	Diet, PA
Bian et al. (43)	At-risk for DM	6	15–1,240	app, dig plat, website		Yes	Diet, PA
Bossen et al. (44)	Patients (CD)	7	22–463	dig plat, mon dev, website	>1	No	PA
Brickwood et al. (45)	General	28	20–470	app, dig plat, mon dev, website	>1	Yes	PA
Brors et al. (46)	Patients (CVD)	21	46–330	app, dig plat, mon dev, website	>1	No	PA
Cavero-Redondo et al. (47)	General	17	25–248	app, dig plat, mon dev, website	>1	Yes	Diet, PA
Chea Tham et al. (48)	General	25	18–544	app, mon dev	>1	No	Diet, PA
Christiansen et al. (49)	At-risk (post partum)	8	18–371	app, digital games, dig plat, email, mon dev, social media, website	>1	No	Diet
Cotter et al. (50)	Patients (DM)	8	35–761	dig plat	SoC	No	Diet, PA
Covolo et al. (51)	General	40	17–1,932	app, PC, mon dev, social media, website	>1	No	Diet, SU, PA
Daryabeygi-Khotbehsara et al. (35)	General	2	17–64	app	SoC	No	PA
Devi et al. (52)	Patients (CVD)	11	15–330	app, website	SoC	Yes	Diet, PA
Duan et al. (53)	Patients (CVD, cancer, chronic respiratory diseases, DM)	15	59–683	app, mon dev, website	>1	Yes	Diet, PA
Dutton et al. (54)	General	18	34–481	website	>1	No	Diet
El Khoury et al. (55)	Patients (obesity, CVD, DM)	22	17–339	app, mon dev, website	>1	Yes	Diet
Haberlin et al. (56)	Patients (cancer)	7	16–206	app, email, website	>1	No	PA
Halldorsdottir et al. (57)	Patients (CVD)	17	74–790	app, email, website	>1	No	Diet, SU, PA
Hardeman et al. (37)	All	6	6–256	app, PC, mon dev	SoC	No	PA
Hayba et al. (58)	At-risk -overweight/obese	6	range N/A; total 7,321	app, dig plat	SoC	No	Diet, PA
Huang et al. (59)	Patients (CVD)	9	30–525	app, PC, dig plat, email	SoC	Yes	SU, PA, social, stress
Hutchesson et al. (60)	General/At-risk -overweight/obese	84	20–2,862	app, PC, digital games, email, mon dev, website	>1	Yes	Diet, PA
Hwang et al. (61)	Patients (cancer survivors)	12	18–556	app, website	SoC	No	Diet, PA, sleep
Jin et al. (62)	Patients (CVD)	30	range N/A; total 7,283	app, PC, dig plat, email	SoC	Yes	Diet, SU, PA, stress
Joiner et al. (63)	General	13	12–220	app, PC, email, mon dev, website	SoC	Yes	Diet, PA
Kelly et al. (64)	Patients (CD)	25	range N/A; total 7,384	app, PC, dig plat, email, website	SoC	Yes	Diet
Khoo et al. (65)	Patients (cancer survivors)	16	30–284	app, mon dev	>1	No	PA
Kim et al. (66)	At risk for metabolic syndrome	18	22–1,032	email, website	SoC	Yes	Diet, PA
Klassen et al. (67)	General/At-risk -overweight/obese	9	range N/A; total 3,821	dig plat, social media, website	>1	No	Diet
Kodama et al. (68)	At-risk -overweight/obese	23	38–2,862	mon dev, website	>1	Yes	Diet, PA
Kuo et al. (69)	Patients- metabolic disorder	21	30–294	app, PC, dig plat, email, mon dev, website	>1	Yes	Diet, PA, stress
Lee et al. (70)	General	12	61–566	app, email, mon dev	>1	No	Diet, PA
Levine et al. (71)	Patients - primary care	16	70–2,862	app, PC,mon dev, website	>1	No	Diet, PA
Lewis et al. (72)	General	11	24–544	app, PC, email, mon dev	>1	No	Diet, PA
Li et al. (73)	All	29	20–935	mon dev	SoC	Yes	PA
Lunde et al. (74)	Patients (CVD)	7	30–519	App	>1	No	Diet, PA, sleep, stress
Lyzwinski et al. (75)	General	21	26–321	app, PC	>1	No	Diet, PA, social stress
Ma et al. (76)	Patients (CVD)	14	44–778	app, PC, website	SoC	Yes	Diet, SU, PA, stress
McCarroll et al. (77)	General	23	17–856	app, email, website	>1	No	Diet, PA
Merriel et al. (78)	At-risk CVD	13	146–3,382	app, dig plat, email, website	SoC	Yes	Diet, SU, PA
Michaud et al. (79)	Patients (DM)	17	range N/A; total 2,212	app, email, mon dev, website	SoC	Yes	Diet, PA
Monninghoff et al. (36)	All	117	15–1,442	app, dig plat, mon dev, social media, website	>1	Yes	PA
Muller et al. (38)	General/At-risk for DM	14	22–69,219	app, PC, digital games, dig plat, email, mon dev, social media, website	>1	No	Diet, PA
Oosterveen et al. (80)	General	45	18–1,698	app, PC, email, mon dev, website	>1	Yes	Diet, SU, PA
Palacios et al. (81)	Patients (CVD)	7	67–562	app, dig plat, email, website	>1	No	Diet, PA, stress
Park et al. (82)	Patients (CVD)	19	6–710	app, email, mon dev, website	>1	No	Diet, PA, stress
Pfaeffli et al. (83)	Patients (CVD)	7	69–521	app, PC, website	SoC	No	Diet, PA
Pradal-Cano et al. (84)	All	14	40–301	app, mon dev	>1	No	PA
Podina and Fodor (85)	At-risk -overweight/obese	47	range N/A; total 15,349	app, PC, mon dev, website	>1	Yes	Diet
Rocha and Kim (86)	General	14	49–883	app, PC, digital games, website	>1	Yes	Diet
Ryan et al. (87)	General	5	52–2,862	app, PC, email, social media, website	>1	No	Diet, PA
Schoeppe et al. (88)	General	19	17–502	app, email, website	>1	No	Diet, PA
Semper et al. (89)	General	4	20–212	app	>1	No	Diet
Seo and Niu (90)	General	31	21–1,692	dig plat, email, social media, website	>1	Yes	Diet
Short et al. (91)	All	12	194–2,827	PC	>1	No	PA
Stevenson et al. (92)	Patients (CKD)	43	6–2,199	app, PC, email, mon dev, website	>1	Yes	Diet, PA
Su et al. (93)	Patients (CVD)	14	15–330	app, PC, email, mon dev, social media, website	>1	Yes	Diet, SU, PA, social sleep
Taylor et al. (94)	All	67	66–11,969	PC, dig plat, email, social media, website	>1	Yes	SU
Tighe et al. (95)	Patients (CVD, chronic respiratory diseases, DM)	5	54–1,325	dig plat	Not described	No	Diet, SU, PA
Tong et al. (96)	All	20	17–977	app, email, mond dev, website	>1	Yes	Diet, SU, PA
Turan et al. (97)	Patients (CVD)	4	28–1,347	app, dig plat, mon dev, website		No	Diet, SU, PA
Van Rhoon et al. (37)	At-risk population	9	22–163	app, email, website	>1	No	Diet, PA
Vegting et al. (98)	Patients (CVD)	9	15–778	email, website	SoC	No	Diet, SU, PA
Villinger et al. (99)	All	27	10–883	Email		Yes	Diet
Wesselman et al. (100)	General/Patients/At-risk (neurocognitive) population	14	Not described	app, website		Yes	Diet, SU, PA, social sleep stress
Wieland et al. (101)	At-risk (overweight or obese)	18	19–1,032	PC, email	>1	Yes	Diet, PA
Williams et al. (102)	General	22	11–3,935	social media	>1	Yes	Diet, PA
Willmott et al. (103)	General	22	12–2,621	app, PC, dig plat, email	>1	No	Diet, PA
Wu et al. (104)	Patients/at-risk DM	16	13–130	App		Yes	Diet, PA
Xu et al. (105)	Patients/hypertension	8	50–443	app, dig plat, social media	>1	Yes	SU, PA

*CD, chronic disease; DM, diabetes; CVD, cardiovascular disease; CKD, chronic kidney disease; Dig Pla, digital platform; PC, personal computer/tablet; mon dev, monitoring devices; PA, physical activity; SU, Substance Use; Social, social relationships; Stress, stress management*.

Of the 72 included reviews, only one evaluated all six lifestyle domains (i.e., diet, PA, SU, social relationships, sleep, and stress management) (100). In this study, the authors evaluated 14 web-based lifestyle programs designed to improve brain health among healthy individuals. A meta-analysis was performed including three RCTs. It showed a small-to-medium positive effect but high heterogeneity.

One systematic review evaluated five lifestyle domains (diet, PA, SU, social relationships, and sleep) (93). The effects of eHealth cardiac rehabilitation on health outcomes were evaluated in 15 trials, and meta-analyses were performed with data from 11 studies. Results indicated that interventions were effective in engaging patients into an active lifestyle, improving quality of life (QoL), and decreasing re-hospitalization. The authors also highlighted that empowerment components and tele-monitoring were crucial to the success of the interventions.

Five systematic reviews evaluated 4 lifestyle domains (59, 62, 74–76): all of them evaluated PA and stress management, 4 evaluated diet, 3 evaluated SU, 2 evaluated social relationships, and 1 evaluated restorative sleep. Most of the studies (*n* = 4) were related to cardiovascular disease (CVD), and 3 of them performed meta-analyses (59, 62, 76). All meta-analyses showed that DHIs are promising methods for managing CVDs.

Fourteen systematic reviews evaluated 3 lifestyle domains and all of them included diet and PA. Nine evaluated SU, 3 evaluated stress management, and 1 evaluated sleep. Only five conducted meta-analyses. These meta-analyses concluded that there is insufficient evidence on the effectiveness of DHI for reducing overall CVD (78). DHI significantly improved physical activity, HbA1c levels, body weight, empowerment, QoL. DHIs were effective, particularly in the short-term, to decrease the number of drinks consumed/week, DHIs may improve healthy behaviors (i.e., PA and healthy diet) but did not appear to reduce unhealthy behaviors (i.e., smoking, alcohol, and unhealthy diet) (39).

The remaining 52 systematic reviews evaluated only one or two domains. Fifty-one evaluated either diet, PA, or their combination (24 presented meta-analyses) and one evaluated DHI for smoking cessation.

### What Are the Most Frequent Lifestyle Domains Addressed by DHI?

**Figure 2:**
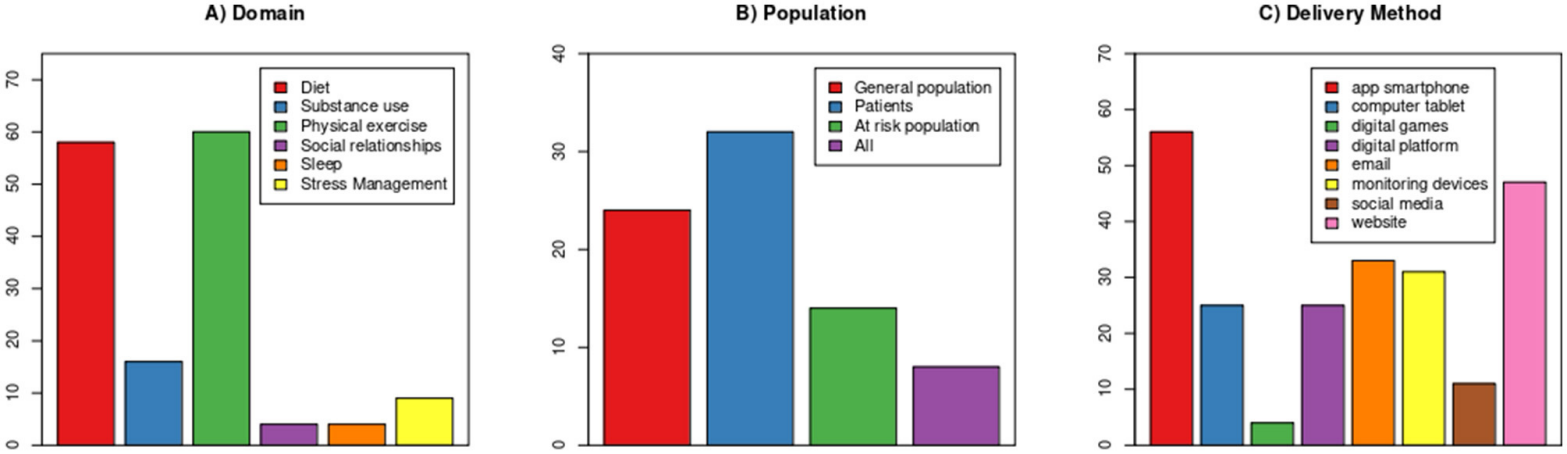
Frequency of systematic reviews that evaluated: **(A)** each lifestyle domain (physical activity, diet/nutrition, substance use, social relationships, sleep, and stress management); **(B)** each population; and **(C)** each delivery method.

Figure 2 shows that the most evaluated domains are Diet/Nutrition (*n* = 58) and PA (*n* = 60), while sleep and social relationships are the least evaluated. Interventions targeting clinical populations were the most frequent (*n* = 32).

### What Are the Most Frequent Outcomes Used to Measure Lifestyle Changes?

**Figure 3:**
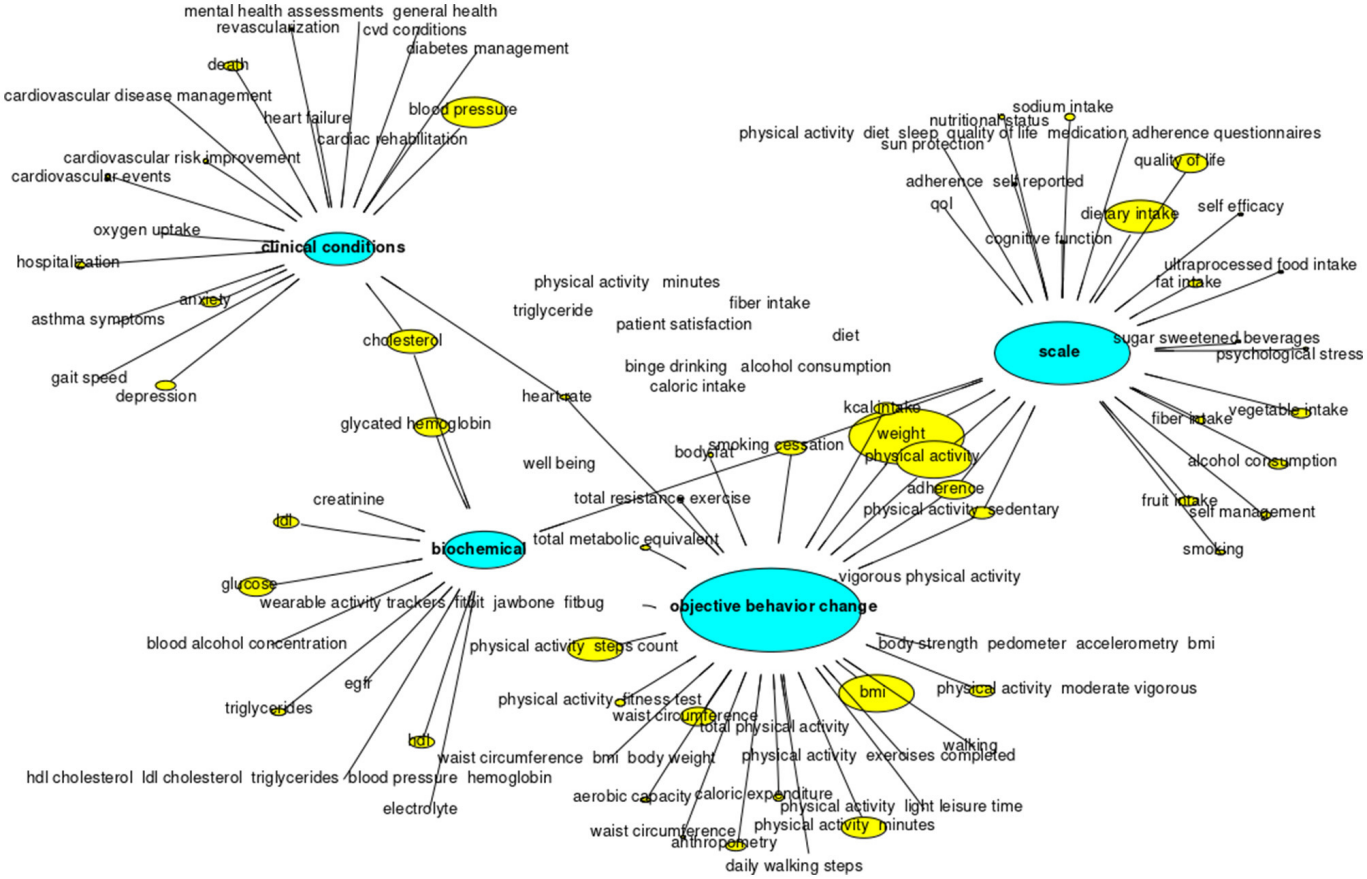
Outcomes evaluated.

Figure 3 shows the outcome groups (i.e., objective measures, self-reported measures, biochemical exams, and clinical conditions) and the description of the outcomes, by their frequency. Considering all the groups, objective measures were the most frequently reviewed, in particular, weight, body mass index (BMI), minutes of physical activity (either per day or week), and steps count. The second group was self-reported measures, specifically using scales/questionnaires to evaluate physical activity and dietary intake. Among biochemical measures, the most frequently reviewed were blood lipids (50, 57, 59, 62, 76, 78, 93, 99) and measures related to DM [such a glucose and glycated hemoglobin (39, 42, 51, 78, 79, 84, 104)]. Finally, regarding clinical conditions, CVD/events (41, 52, 59, 62, 80–82, 92, 93, 95, 96) and psychiatric disorders [depression (46, 80, 81, 84, 87, 92, 93) and anxiety (59, 80, 81, 84, 92, 93)] were the most common. Some outcomes, such as smoking cessation, were reviewed either as objective measures (94) or self-reported (39, 79, 80, 83, 93–97, 105).

### What Are the Most Frequent DHI Delivery Methods?

The most frequent delivery methods of DHIs were apps (*n* = 47) and websites (*n* = 42), followed by e-mails and monitoring devices (Figure 2). Notably, from the 72 studies included, 62 evaluated the effectiveness of more than one delivery method.

## Discussion

To our knowledge, this is the first scoping review mapping the most frequent lifestyle domains and outcomes addressed by DHIs. Up to September 2021, we observed that only one systematic review simultaneously evaluated the six lifestyle domains proposed by the American College of Lifestyle Medicine (12). The majority of the reviews targeted diet and physical activity. Objective measures, such as weight and BMI, were the most frequently observed outcomes, followed by self-reported questionnaires. DHIs were delivered using different technologies, with smartphone apps and websites being the most common.

Lifestyle is a developing research theme, which has exponentially attracted interest during the COVID-19 pandemic. Researchers around the world are evaluating the delayed effect of unhealthy behaviors during the pandemic over the population health (i.e., increased incidence of chronic diseases and mental health problems after the changes in lifestyle). Overall, studies evaluated only one or a couple of behaviors that may have changed during this period (106, 107). Such finding is convergent with our results, showing that most reviews evaluated a single behavior/domain or a combination of diet and PA. This approach is valuable for estimating prevalence and specific targets for public policies, as well as increasing our understanding of each specific domain. However, at the individual level, it disregards that a healthy lifestyle represents a complex balance among multiple behaviors and that the development of chronic diseases results from the combination of different risk factors (i.e., genetic, lifestyle, and environmental) (108). LM, relying on six main domains, may be a step forward to address those behaviors, their complex interactions, and interconnections—especially if a multidimensional approach is applied. From a research perspective, the main priority should be to clarify the concept of lifestyle, and how to operationalize it. In this sense, guidelines and reporting guidelines are still missing and could be of great value to inform further studies.

From the 72 systematic reviews included in our study, 58 addressed diet/nutrition, and 60 evaluated PA. Diet and PA are fundamental features of a healthy lifestyle and well-known risk factors for chronic diseases (1, 11, 109). Given this importance, it is not a surprise that the most frequent domains and outcomes evaluated in the literature were related to them. Additional factors that may explain the frequency of DHIs addressing diet/PA include their importance for clinical populations, and the availability of objective outcomes to be measured (such as weight, BMI, and biochemical measures). Still, the high heterogeneity of those outcomes, delivery methods, and control groups precluded most of the systematic reviews to perform meta-analyses on the effectiveness of the DHI. Beyond the consensus on those aspects, an upcoming challenge is if and how hard outcomes (mortality or stroke for example) will ever be potential targets for DHIs. Hard outcomes take a longer follow-up time to be observed, while DHIs are fast-changing and volatile. It is possible we will need “adaptive” interventions, with real-time evaluation and modifications guided by short-term outcomes. Although methodological and ethical issues will be raised, creating new short-lived interventions will hardly be cost-effective and/or sustainable in the long run.

Regarding SU, LM literature concentrates on the use of tobacco and alcohol which, at the population level, present the highest impact on health. Overall, 7.7 million deaths were attributable to smoking in 2019, with smoking being the major cause of death among men in the world (110). Since 2015, it is recommended that clinicians screen all adults for cigarette smoking and prescribe some behavioral and pharmacotherapy therapy for smokers (unless there is some medical contraindication) (111). In 2016, there were 2.4 billion current drinkers globally, and 2.8 million deaths were attributed to alcohol (112). The burden of disease related to alcohol use may be seen as a result of binge drinking and alcohol use disorders, which usually require different treatment/prevention approaches. Given such figures, it is remarkable that we found only sixteen reviews addressing DHI to decrease SU (mostly to reduce smoking). It is possible that researchers do not recognize the importance of these substances or do not have sufficient training to provide evidence-based interventions. In fact, alcohol use disorders are among the least diagnosed medical conditions in clinical practice (113), and alcohol remains the blind spot of global health (114). In addition, as in the case of diet and PA, most reviews evaluating SU were conducted among clinical or at-risk populations, which may represent the most severe and least responsive individuals. Nevertheless, a recent review of systematic reviews found that DHIs have a small but positive effect to decrease alcohol and tobacco use among the general population (115). Such results are encouraging in the sense that DHI, together with public policies, may represent a step forward in the primary prevention of more than 200 diseases caused by smoking and/or alcohol use. One important aspect to evolve in this endeavor is to rigorously evaluate the severity of the SU disorder to determine who is going to benefit and which components of the DHIs are effective.

Stress management and social relationships were among the least evaluated lifestyle domains in our study. Compared to diet and PA, social relationships and stress management present less evidence of the effect on hard outcomes such as mortality. Furthermore, it is difficult to find reliable objective measures/outcomes to be used in DHI. Innovative digital methods to address this gap are necessary and these areas could benefit from passive sensing data obtained *via* smartphones and wearable devices, in the same way, step count is used to evaluating PA. Digital phenotyping through these tools allows quantifying the biological *stress* response in real time (116). In experimental conditions, algorithms developed based on physiological proxies of the autonomic nervous system activity (e.g., heart rate, body temperature, skin temperature, and conductance) have demonstrated a high accuracy to detect stress (117). Moreover, smartphone-based episodic audio recordings allow analyses of voice and speech features that may be potential vocal markers of stress (118, 119). Phone usage and the number of calls, texts, and interactions in online social media can be used as proxies of *social* contact or interaction (116, 120–122), while global positioning system (GPS)-based mobility and conversations captured by smartphone microphones were used to estimate social activity and loneliness in individuals with schizophrenia (123). Social support, however, is more complex than social contacts. To also account for the quality of social relationships, calls to close contacts with strong ties (such as family members and close friends) have been evaluated (124). Moreover, preliminary evidence suggests that passive audio collection on smartphones combined with machine learning techniques helped to identify auditory stimuli from the social environment of adolescents and young mothers that can signal social support (125).

In the same manner, sleep was evaluated in only three reviews. Sleep problems are a growing concern in global public health due to the severe consequences of poor sleep on cognition, emotion, the risk for serious medical conditions, and mortality (126). Particularly during the COVID-19 pandemic, sleep problems have been highly prevalent with ~40% of the general population reporting poor sleep quality (127). Sleep comprises of different areas, such as sleep onset latency, sleep restfulness, sleep disturbances, sleep schedule, efficiency, and daytime napping, among others (126, 128). Traditional, non-digital interventions to improve sleep quality are well-established and have been used to target specific problems, such as cognitive-behavioral therapy (CBT), relaxation and mindfulness therapy, stimulus control therapy, sleep hygiene education, and similar cognitive-behavioral interventions aiming to improve these areas (129, 130). DHIs for sleep improvements, such as digital CBT and digital sleep restriction therapy, have demonstrated potential as effective e-health interventions, however, further refinement and investigations of these delivery methods are required (131, 132).

Digital Health Interventions have emerged as promising methods to help patients and people at risk by providing immediate access to suggestions on improvement of nutritional and PA habits, education about their conditions, and medication adherence. The most frequent delivery method we found was the use of a smartphone, which has increased over the last decade following the widespread of this technology. The progressive advancement of efficient, accessible, and reliable technology has made it possible for various populations to have quick access to information and health. This progress has led to the exponential increase of the number of apps addressing lifestyle (22, 133), and the COVID-19 pandemic will likely lead to a further increase in those numbers. Beyond issues regarding costs (a considerable proportion of apps is paid, although it is hard to estimate the exact proportion) and accessibility (in populations with low cell phone coverage), adherence, usability, and long-term engagement remain to be improved. Co-design and user experience design are important ways to overcome these barriers, as well as behavioral sciences and health economics (134). As mentioned before, the proper scientific evaluation of DHI effectiveness is still to be determined but important steps, such as the publication of the NICE framework (27) and the WHO recommendations (21) have advanced the field. Importantly, while RCTs are the gold standard to evaluate DHIs, the CONSORT EHEALTH extension should be widely adopted to improve their quality, transparency, and reproducibility (135). In the same direction, systematic reviews and meta-analyses should also evaluate how closely are those guidelines being followed by authors. Another aspect that has not been adequately assessed in the systematic reviews is the possibility of harm, including confidentiality breaches. Although most people may believe DHIs present low-level harm, it is still necessary to report on that, and even bring to light harms that were not considered beforehand.

Our study has some limitations, such as the absence of data (e.g., the sample size of included RCTs) for some systematic reviews, and the complexity of study characteristics: different populations (general population and people at risk or living with specific diseases), interventions, delivery methods, and comparators. To properly answer our research question, the inclusion criteria were broad, including as many systematic reviews as possible, which limited synthesis and comparisons between studies. However, scoping reviews usually aim to provide an overview/map of the evidence instead of the result/answer to a particular question (136). Nevertheless, our review highlighted the following major gaps to be addressed regarding DHIs to improve lifestyle: (1) the concept of lifestyle is unclear and fragmented, including mostly one or two unhealthy behaviors; (2) lifestyle domains for which there are few objective outcomes to be measured (such as SU, social relationships, and stress management) are understudied; (3) there is a lack of assessment of hard/long-term outcomes; (4) although DHIs are valued by their high accessibility, most studies are not designed to include the general population; and (5) there is high heterogeneity on reporting the methods, such as outcomes, description of the interventions, and control groups.

The COVID-19 pandemic has shown the importance of connections and real-time data monitoring in health (137) and has consolidated the use of technology (such as tele-medicine) across the globe. DHIs will be increasingly important to improve lifestyle at primary, secondary, and tertiary prevention. However, to be cost-effective and sustainable, such interventions will need to be constantly monitored, adapted, and fully integrated into health systems. Integrating technologies and data are crucial to inform precision medicine, allowing algorithms to suggest the best interventions for each individual. LM and Public Health may profit from those advances to prevent chronic diseases.

## Data Availability Statement

The original contributions presented in the study are included in the article/Supplementary Material, further inquiries can be directed to the corresponding author/s.

## Author Contributions

RC and RD conceived the study. RC and CR searched the literature. RC, HP, CO, CR, MS, and RD reviewed the literature and extracted data. MR-A analyzed data. RC, MS, and RD wrote the first draft. VB-M and FK reviewed for important intellectual content. All authors contributed to the article and approved the submitted version.

## Funding

RD would like to thank the Conselho Nacional de Desenvolvimento Científico e Tecnologico (CNPq #312543/2020-4) and Fundação de Amparo ‘a Pesquisa do Estado do Rio de Janeiro (FAPERJ E-26/203.154/2017).

## Conflict of Interest

The authors declare that the research was conducted in the absence of any commercial or financial relationships that could be construed as a potential conflict of interest.

## Publisher's Note

All claims expressed in this article are solely those of the authors and do not necessarily represent those of their affiliated organizations, or those of the publisher, the editors and the reviewers. Any product that may be evaluated in this article, or claim that may be made by its manufacturer, is not guaranteed or endorsed by the publisher.
