# Supplementary material for: What Are We Measuring When We Evaluate Digital Interventions for Improving Lifestyle? A Scoping Meta-Review
Source: Front Public Health. 2022 Jan 3;9:735624. doi: 10.3389/fpubh.2021.735624 (PMC8761632; doi:10.3389/fpubh.2021.735624)
Supplement: Supplementary file 1 [file Table_1.DOCX]

**Supplementary Material 1**

**Search activities**

**Pubmed search strategy (n = 166); Updated: 28/10/2021**

("life style"[MeSH Terms] OR "life styles"[Title/Abstract] OR "Lifestyle"

[Title/Abstract] OR "Lifestyles"[Title/Abstract] OR (("life style"[MeSH Terms] OR

("Life"[All Fields] AND "style"[All Fields]) OR "life style"[All Fields]) AND "induced

illness"[Title/Abstract])) AND ("e-health"[Title/Abstract] OR "ehealth"

[Title/Abstract] OR "mobile health"[Title/Abstract] OR "app"[Title/Abstract] OR

"internet"[Title/Abstract] OR "smartphone"[Title/Abstract] OR "mhealth"

[Title/Abstract] OR "mobile health"[Title/Abstract] OR "web based"[Title/Abstract] OR

"telehealth"[Title/Abstract] OR "social media"[Title/Abstract])

**Web of Science search strategy (n = 544); Updated: 28/10/2021**

Life Style* OR Lifestyle* OR Life Style Induced Illness) AND (e-health OR ehealth OR mobile Health OR app OR internet OR smartphone OR mhealth OR mobile health OR web based OR telehealth OR social media) AND (systematic review OR meta-analysis OR metanalysis)

**Embase search strategy (n = 189); Updated: 28/10/2021**

**#1)** (**'life'** OR **'life'**/exp OR **life**) AND style$ OR lifestyle$

**#2)** ((((**'e health'**/exp OR **'e health'** OR **'ehealth'**/exp OR **ehealth** OR **mobile**) AND (**'health'**/exp OR **health**) OR **app** OR **'internet'**/exp OR **internet** OR **'smartphone'**/exp OR **smartphone** OR **'mhealth'**/exp OR **mhealth** OR **mobile**) AND (**'health'**/exp OR **health**) OR **'web'**/exp OR **web**) AND **based** OR **'telehealth'**/exp OR **telehealth** OR **'social'**/exp OR **social**) AND (**'media'**/exp OR **media**)

**#3) systematic** AND (**'review'**/exp OR **review**) OR **'meta analysis'**/exp OR **'meta analysis'** OR **metanalysis**

**#4) #1** AND **#2** AND **#3**

**#5) #4** AND (**2010**:py OR **2011**:py OR **2012**:py OR **2013**:py OR **2014**:py OR **2015**:py OR **2016**:py OR **2017**:py OR **2018**:py OR **2019**:py OR **2020**:py OR **2021**:py)

**Cochrane library search strategy (n = 54); Updated: 28/10/2021**

(Life Style* OR Lifestyle* OR Life Style Induced Illness) AND (e-health OR ehealth OR mobile Health OR app OR internet OR smartphone OR mhealth OR mobile health OR web based OR telehealth OR social media) AND (systematic review OR meta-analysis OR metanalysis) in Title Abstract Keyword

**List of excluded papers with reasons**

| **Title** | **Year** | **Journal** | **Reason** |
| --- | --- | --- | --- |
| Characteristics of effective Internet-mediated interventions to change lifestyle in people aged 50 and older: a systematic review. | 2011 | Ageing research reviews | wrong population |
| Psychosocial interventions for patients and caregivers in the age of new communication technologies: opportunities and challenges in cancer care. | 2015 | Journal of health communication | wrong study design |
| Effectiveness of Mobile Health Interventions Promoting Physical Activity and Lifestyle Interventions to Reduce Cardiovascular Risk Among Individuals With Metabolic Syndrome: Systematic Review and Meta-Analysis. | 2020 | Journal of medical Internet research | wrong study design |
| High-tech tools for exercise motivation: Use and role of technologies such as the internet, mobile applications, social media, and video games | 2015 | Diabetes Spectr. | wrong study design |
| A narrative synthesis systematic review of digital self-monitoring interventions for middle-aged and older adults | 2019 | INTERNET INTERVENTIONS-THE APPLICATION OF INFORMATION TECHNOLOGY IN MENTAL AND BEHAVIOURAL HEALTH | wrong study design |
| Effectiveness of lifestyle-based weight loss interventions for adults with type 2 diabetes: a systematic review and meta-analysis | 2015 | DIABETES OBESITY & METABOLISM | wrong intervention |
| The use of social features in mobile health interventions to promote physical activity: a systematic review. | 2018 | NPJ digital medicine | wrong study design |
| eHealth for improving quality of life in breast cancer patients: A systematic review. | 2019 | Cancer treatment reviews | wrong outcome |
| eHealth as the Next-Generation Perinatal Care: An Overview of the Literature | 2018 | JOURNAL OF MEDICAL INTERNET RESEARCH | wrong population |
| Web-Based Tools for Text-Based Patient-Provider Communication in Chronic Conditions: Scoping Review | 2017 | JOURNAL OF MEDICAL INTERNET RESEARCH | wrong outcome |
| Lifestyle modification for metabolic syndrome: a systematic review. | 2014 | The American journal of medicine | wrong intervention |
| Attenuating Pregnancy Weight Gain-What Works and Why: A Systematic Review and Meta-Analysis. | 2018 | Nutrients | wrong population |
| A Systematic Review of Application and Effectiveness of mHealth Interventions for Obesity and Diabetes Treatment and Self-Management | 2017 | ADVANCES IN NUTRITION | wrong population |
| The effect of smartphone application interventions on physical activity level among university/college students: a systematic review protocol | 2020 | PHYSICAL THERAPY REVIEWS | wrong study design |
| Chinese Cardiovascular Disease Mobile Apps' Information Types, Information Quality, and Interactive Functions for Self-Management: Systematic Review. | 2017 | JMIR mHealth and uHealth | wrong study design |
| Effects of Mobile Health App Interventions on Sedentary Time, Physical Activity, and Fitness in Older Adults: Systematic Review and Meta-Analysis. | 2019 | Journal of medical Internet research | wrong population |
| Web-Based Interventions Targeting Cardiovascular Risk Factors in Middle-Aged and Older People: A Systematic Review and Meta-Analysis. | 2016 | Journal of medical Internet research | wrong population |
| mHealth technology for ecological momentary assessment in physical activity research: a systematic review. | 2020 | PeerJ | wrong outcome |
| Effectiveness of Smartphone-Based Physical Activity Interventions on Individuals' Health Outcomes: A Systematic Review. | 2021 | BioMed research international | wrong population |
| Digital technology to support lifestyle and health behaviour changes in surgical patients: systematic review. | 2021 | BJS open | wrong study design |
| Games and Health Education for Diabetes Control: A Systematic Review with Meta-Analysis. | 2020 | Healthcare (Basel, Switzerland) | wrong intervention |
| Impact of technology-based patient education on modifiable cardiovascular risk factors of people with coronary heart disease: A systematic review. | 2020 | Patient education and counseling | wrong intervention |
| Digital Health Coaching Programs Among Older Employees in Transition to Retirement: Systematic Literature Review. | 2020 | Journal of medical Internet research | wrong population |
| Current Evidence and Directions for Future Research in eHealth Physical Activity Interventions for Adults Affected by Cancer: Systematic Review | 2021 | JMIR CANCER | wrong study design |
| Promoting healthy lifestyles using information technology during the COVID-19 pandemic | 2021 | REVIEWS IN CARDIOVASCULAR MEDICINE | wrong study design |
| Intervention programs through the Internet for weight loss in adults with overweight and obesity: a systematic review | 2020 | REVISTA ESPANOLA DE NUTRICION HUMANA Y DIETETICA | no data |
| Telemedicine intervention-reduced blood pressure in a chronic disease population: A meta-analysis |  | JOURNAL OF TELEMEDICINE AND TELECARE | no data |
| Internet-Based Interventions in Chronic Somatic Disease | 2018 | DEUTSCHES ARZTEBLATT INTERNATIONAL | wrong study design |
| Community-based efforts to promote physical activity: a systematic review of interventions considering mode of delivery, study quality and population subgroups. | 2014 | Journal of science and medicine in sport | wrong intervention |
| Information Technology and Lifestyle: A Systematic Evaluation of Internet and Mobile Interventions for Improving Diet, Physical Activity, Obesity, Tobacco, and Alcohol Use. | 2016 | Journal of the American Heart Association | wrong study design |
| Effect of web-based tailored lifestyle interventions on fruit and vegetable consumption in adults: A systematic review and meta-analysis of randomised controlled trials | 2015 | PROCEEDINGS OF THE NUTRITION SOCIETY | wrong study design |
| The use of social media in nutrition interventions for adolescents and young adults-A systematic review. | 2018 | International journal of medical informatics | wrong population |
| [Effectiveness of lifestyle apps not yet proven]. | 2016 | Nederlands tijdschrift voor geneeskunde | no data |
| Effectiveness, acceptability and usefulness of mobile applications for cardiovascular disease self-management: Systematic review with meta-synthesis of quantitative and qualitative data. | 2018 | European journal of preventive cardiology | wrong study design |
| Use of mobile phones as a tool for weight loss: a systematic review. | 2014 | Journal of telemedicine and telecare | wrong study design |
| Evidence on the Use of Mobile Apps During the Treatment of Breast Cancer: Systematic Review | 2019 | JMIR MHEALTH AND UHEALTH | wrong outcome |
| [Can apps encourage a healthier and more active lifestyle?]. | 2016 | Nederlands tijdschrift voor geneeskunde | wrong study design |
| Behavior Change Techniques in mHealth Apps for the Mental and Physical Health of Employees: Systematic Assessment | 2018 | JMIR MHEALTH AND UHEALTH | wrong study design |
| Mobile health applications for chronic diseases: A systematic review of features for lifestyle improvement. | 2019 | Diabetes & metabolic syndrome | wrong study design |
| mHealth Technologies to Influence Physical Activity and Sedentary Behaviors: Behavior Change Techniques, Systematic Review and Meta-Analysis of Randomized Controlled Trials. | 2017 | Annals of behavioral medicine : a publication of the Society of Behavioral Medicine | wrong population |
| Mobile Health Applications in Weight Management: A Systematic Literature Review. | 2019 | American journal of preventive medicine | wrong study design |
| The efficacy of electronic health interventions targeting improved sleep for achieving prevention of weight gain in adolescents and young to middle-aged adults: A systematic review | 2020 | OBESITY REVIEWS | wrong population |
| Patients' Use of Social Media for Diabetes Self-Care: Systematic Review | 2020 | JOURNAL OF MEDICAL INTERNET RESEARCH | wrong study design |
| Health Economic Evaluations of Digital Health Interventions for Secondary Prevention in Stroke Patients: A Systematic Review | 2019 | CEREBROVASCULAR DISEASES EXTRA | no data |
| Emerging technology in promoting physical activity and health: Challenges and opportunities | 2019 | J. Clin. Med. | wrong study design |
| Online and mobile technologies for self-management in bipolar disorder: A systematic review. | 2017 | Psychiatric rehabilitation journal | wrong study design |
| Telephone, print, and Web-based interventions for physical activity, diet, and weight control among cancer survivors: a systematic review. | 2015 | Journal of cancer survivorship : research and practice | wrong study design |
| The Internet and the therapeutic education of patients: A systematic review of the literature. | 2010 | Annals of physical and rehabilitation medicine | wrong intervention |
| Behavioural weight management programmes for adults assessed by trials conducted in everyday contexts: systematic review and meta-analysis | 2014 | OBESITY REVIEWS | no data |
| Effectiveness of Social Media-based Interventions on Weight-related Behaviors and Body Weight Status: Review and Meta-analysis | 2017 | AMERICAN JOURNAL OF HEALTH BEHAVIOR | wrong study design |
| The use of telemedicine for delivering healthcare in Japan: Systematic review of literature published in Japanese and English languages | 2017 | JOURNAL OF TELEMEDICINE AND TELECARE | wrong outcome |
| Using Digital Health Technology to Prevent and Treat Diabetes | 2020 | Diabetes Technol. Ther. | wrong study design |
| IoT-based systems for improving older adults' wellbeing: a systematic review | 2019 | 2019 14TH IBERIAN CONFERENCE ON INFORMATION SYSTEMS AND TECHNOLOGIES | wrong population |
| Digital Health Technologies to Promote Lifestyle Change and Adherence | 2017 | Curr. Treat. Options Cardiovasc. Med. | wrong study design |
| The Impact of Automated Brief Messages Promoting Lifestyle Changes Delivered Via Mobile Devices to People with Type 2 Diabetes: A Systematic Literature Review and Meta-Analysis of Controlled Trials. | 2016 | Journal of medical Internet research | wrong intervention |
| Consideration of intelligent applications to support diabetic patients: A scoping review for nutrition mobile phone apps | 2020 | JOURNAL OF CONTEMPORARY MEDICAL SCIENCES | wrong study design |
| Lifestyle behavior interventions delivered using technology in childhood, adolescent, and young adult cancer survivors: A systematic review. | 2017 | Pediatric blood & cancer | wrong population |
| Use of social media platforms for promoting healthy employee lifestyles and occupational health and safety prevention: A systematic review. | 2020 | Safety science | no data |
| Development of a Healthy Lifestyle Mobile App for Overweight Pregnant Women: Qualitative Study | 2018 | JMIR MHEALTH AND UHEALTH | wrong study design |
| Electronic-based lifestyle interventions in overweight or obese perinatal women: a systematic review and meta-analysis | 2017 | OBESITY REVIEWS | wrong population |
| Persuasive System Design Principles and Behavior Change Techniques to Stimulate Motivation and Adherence in Electronic Health Interventions to Support Weight Loss Maintenance: Scoping Review | 2019 | JOURNAL OF MEDICAL INTERNET RESEARCH | wrong study design |
| Video games for diabetes self-management: Examples and design strategies | 2012 | J. Diabetes Sci. Technol. | wrong study design |
| Health Professionals' and Postpartum Women's Perspectives on Digital Health Interventions for Lifestyle Management in the Postpartum Period: A Systematic Review of Qualitative Studies. | 2019 | Frontiers in endocrinology | wrong study design |
| Behavior Change Techniques Implemented in Electronic Lifestyle Activity Monitors: A Systematic Content Analysis | 2014 | JOURNAL OF MEDICAL INTERNET RESEARCH | wrong study design |
| Potential Use of Mobile Phone Applications for Self-Monitoring and Increasing Daily Fruit and Vegetable Consumption: A Systematized Review. | 2019 | Nutrients | wrong population |
| Social Networking Strategies That Aim To Reduce Obesity Have Achieved Significant Although Modest Results | 2014 | HEALTH AFFAIRS | wrong study design |
| Effect of Lifestyle Coaching Including Telemonitoring and Telecoaching on Gestational Weight Gain and Postnatal Weight Loss: A Systematic Review | 2019 | TELEMEDICINE AND E-HEALTH | wrong population |
| Apps to promote physical activity among adults: a review and content analysis | 2014 | INTERNATIONAL JOURNAL OF BEHAVIORAL NUTRITION AND PHYSICAL ACTIVITY | wrong study design |
| Appropriate Interventions for Pregnant Women with Indicators of Metabolic Syndrome on Pregnancy Outcomes: A Systematic Review | 2019 | INTERNATIONAL JOURNAL OF PREVENTIVE MEDICINE | wrong population |
| The effectiveness of distance-based interventions for smoking cessation and alcohol moderation among cancer survivors: A meta-analysis | 2020 | PSYCHO-ONCOLOGY | wrong intervention |
| Addressing preconception behaviour change through mobile phone apps: a protocol for a systematic review and meta-analysis. | 2019 | Systematic reviews | wrong study design |
| A systematic review of weight loss, physical activity and dietary interventions involving African American men | 2014 | OBESITY REVIEWS | wrong intervention |
| A meta-analysis of self-determination theory-informed intervention studies in the health domain: effects on motivation, health behavior, physical, and psychological health | | HEALTH PSYCHOLOGY REVIEW | wrong intervention |
| A framework for examining the function of digital health technologies for weight management | 2018 | TRANSLATIONAL BEHAVIORAL MEDICINE | wrong study design |
| The Usability and Effectiveness of Mobile Health Technology-Based Lifestyle and Medical Intervention Apps Supporting Health Care During Pregnancy: Systematic Review. | 2018 | JMIR mHealth and uHealth | wrong study design |
| Cost-effectiveness of health-related lifestyle advice delivered by peer or lay advisors: synthesis of evidence from a systematic review. | 2013 | Cost effectiveness and resource allocation : C/E | wrong intervention |
| Conceptualising engagement with digital behaviour change interventions: a systematic review using principles from critical interpretive synthesis | 2017 | TRANSLATIONAL BEHAVIORAL MEDICINE | wrong outcome |
| Persuasive features in health information technology interventions for older adults with chronic diseases: a systematic review | 2016 | HEALTH AND TECHNOLOGY | wrong outcome |
| A systematic review on research into the effectiveness of group-based sport and exercise programs designed for Indigenous adults | 2016 | JOURNAL OF SCIENCE AND MEDICINE IN SPORT | wrong intervention |
| Assessment of the Efficacy, Safety, and Effectiveness of Weight Control and Obesity Management Mobile Health Interventions: Systematic Review | 2019 | JMIR MHEALTH AND UHEALTH | wrong study design |
